# Re-audit of the use of flumazenil following midazolam-induced conscious sedation

**DOI:** 10.1038/s41405-023-00163-7

**Published:** 2023-08-04

**Authors:** Muhammad Syafiq Asyraf Rosli, Ellie Heidari

**Affiliations:** 1 Clinical Lecturer, Faculty of Dentistry, National University of Malaysia, Kuala Lumpur, Malaysia; 2 Postgraduate Dentist, Sedation and Special Care Department, Faculty of Dentistry, Oral & Craniofacial Sciences, King’s College London, London, UK; 3 Senior Specialist Clinical Teacher, Centre for Dental Education, Faculty of Dentistry, Oral & Craniofacial Sciences, King’s College London, London, UK

**Keywords:** Dentistry, Special care dentistry

## Abstract

**Introduction:**

Flumazenil is an antagonist drug of Benzodiazepam (BDZ) that has been used as a reversal agent of midazolam-induced conscious sedation (CS) in both emergency and elective procedures. For CS procedure, a high-quality record keeping and clinical justification prior to admission of flumazenil are recommended. Clinical Audit (CA) enables clinicians to evaluate the quality and standard of recorded clinical procedures.

**Methods:**

This re-audit investigated the reasons for the use of flumazenil and record keeping’s quality with particular emphasis on CS. In this retrospective reaudit, the authors looked at the patients’ records who had received dental care under CS in the Sedation and Special Care Department of (SSCD), United Kingdom from January to June 2022.

**Results:**

Out of 665 patients who received midazolam-induced CS, 21 patients were administered IV Flumazenil. The commonest reason (9, 42.8%) was due to prolonged recovery.

**Conclusion:**

This re-audit highlighted the need for administrating flumazenil in certain patient groups, and/or circumstances (not emergency). The importance of maintaining high-quality record keeping is discussed.

## Introduction

Satisfactory pain and anxiety control is an essential components in the practice of clinical dentistry [1]. In the past decades, midazolam has been regarded as a drug of choice for dental procedures due to its wide margin of safety and minimal post-operative complications [2].

Flumazenil, the first specific antagonist drug for Benzodiazepam (BDZ) has been used as a reversal agent of midazolam-induced conscious sedation (CS) in both emergency and elective cases [3]. Flumazenil does not fully antagonise the effect of midazolam with regards to psychomotor or cognitive ability [4]. Therefore, it may lead to residual sedation in theory with the effect of Midazolam (such as anxiolysis, and impaired cognitive function) may re-emerge [5]. Hence why when flumazenil is used, it is important to assess the patient fully and discharge the patient to a fully informed escort.

Flumazenil can play an important role for certain patient groups such as special care patients to help the escort to take the patients home, especially in those with long journeys [6]. The patient case selection for CS, it is delivery by trained members of the team, and post-operative care are important. Intercollegiate Advisory Committee for Sedation in Dentistry (IACSD) and Scottish Dental Clinical Effectiveness Programme [1] guideline has outlined clearly the standards for the provision of dental care under CS [1, 7]. There are several factors that play an important role in achieving optimal levels of CS for patients. Each case should be assessed individually. Factors such as titration regimes can play a role in achieving sedation levels. In cases where midazolam is administered intranasally or orally, it is given as a bolus dose and therefore it is not titrated against a patient’s response. In these cases, it might be a risk of oversedation. The trained team are aware of appropriate actions to take when / if this situation arises.

The National Patient Safety Agency (NPSA)’s Rapid Response Report (RRR) also suggests that the therapeutic use of flumazenil needs to be documented clearly and audited. Additionally, the report highlights the importance of not routinely relying on flumazenil as a reversal agent. However, staff have mentioned that in certain NHS Trusts the use of flumazenil is regarded as a never event and a concern among the Trusts can be that a reduced cost of purchasing flumazenil might influence its use. In any CS audits, the reasons and frequency of flumazenil use can be investigated. The audits’ results can then present an opportunity for the oral health care team to review their practises and compare that to the recommended best practise and standards.

A high-quality record keeping is significantly important for patient’s safety. Additionally, a comprehensive record with relevant information is a regulatory requirement in patient care. It helps with tailored care provision based on the previous useful available information [1, 8]. Maintaining and protecting patients’ information is one of the nine principles set out by the General Dental Council, United Kingdom (GDC UK) [9].

In view of the importance of maintaining high standard CS record keeping and appropriate justification for the use of flumazenil as a reversal agent after midazolam-induced CS, a clinical audit (CA) was conducted in Sedation and Special Care Department (SSCD), Guy’s Hospital, United Kingdom [6]. CA is a systematic approach that evaluates the effectiveness of health care and ensures that the clinical practice is continuously monitored against established standard and any deficiencies in relation to the set standards of care are remedied [10]. It is also one of the main pillars of clinical governance of the National Health Service (NHS UK) [11]. Care Quality Commission, United Kingdom (CQC UK) publishes documentations to prevent the patient from receiving unsafe care and treatment [12]. The regulation has previously highlighted the importance of risk assessment and prescription of sufficient dosage of medicine to ensure the patient safety.

The SSCD receives referrals of approximately 1000 cases per year. The cases can range from medically compromised patients with dental anxiety to in-patient cases. For some patients, the use of CS may facilitate the provision of dental care while for others it can control a possible exacerbation of their current systematic condition (e.g., asthma). In the post-pandemic era, we are seeing an increased number of patients presenting with high oral health needs. These patients have not had access to care for a while and might be anxious about attending dental services again. Limited access to routine dental care during this period (COVID-19) has also contributed to more complex care needed especially among vulnerable patients [13]. Therefore, in view of the mentioned factors, this reaudit cycle was undertaken to:

1. To identify any changes since the first conducted CA [6]
2. To assess the reasons for the use of flumazenil as a reversal agent of midazolam-induced CS in SSCD
3. To assess the quality of record keeping in regard to CS (the reason for the use of flumazenil, the time of administration and the time of discharge)
4. To assess the compliance of clinical staff in SSCD on local standards that was set from previous clinical audit and to identify if any improvements are needed

## Materials and methods

The SSCD provides care for a range of patients considered to have complex medical problems (such as oncology), patients with learning and physical disability. The staff can combine non-pharmacological behavior management techniques with the use of pharmacological interventions to provide dental care. The commonest intravenous (IV) CS agent used is midazolam, where the clinicians titrate the drug according to the patient’s response.

**Fig. 1:**
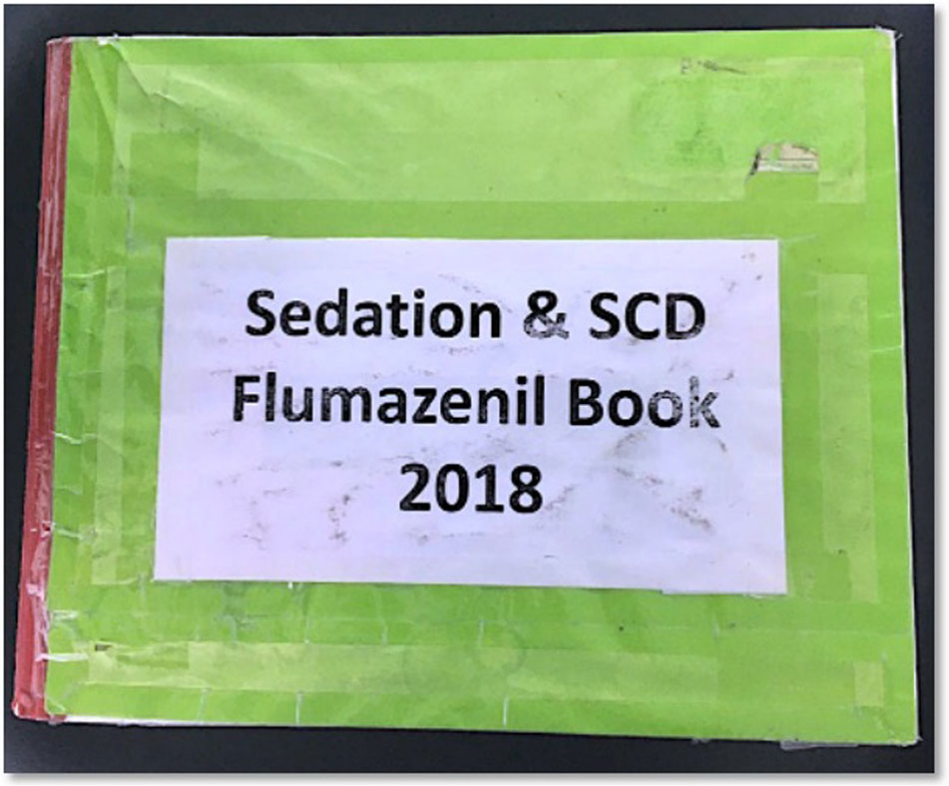
The Sedation and Special Care Department (SSCD) Flumazenil Drug book.

Flumazenil is administered in certain circumstances (such as difficult long journeys home for a patient with mobility difficulties). Once flumazenil has been used, this will be recorded in the SSCD flumazenil drug book (Fig. 1) and the patient’s clinical records. In the drug book, the reason for IV flumazenil use has been classified into seven categories. The categories are emergency, special care patients, to enable and assist escorts for difficult journeys, prolonged recovery, post-op retching and others (e.g, to facilitate dental radiographic procedure).

The recorded data from the SSCD flumazenil drug book and patient’s record was used for this retrospective reaudit (January to June 2022) to identify any changes since the last carried audit (2010) [6]. We identified the reasons for flumazenil use and assessed the quality of electronic patient’s records of those who had flumazenil following midazolam-induced CS.

Table 1 outlines the data that was collected from the Flumazenil drug book and patient’s records.

**Table 1:** Data collected from Flumazenil drug book and patient’s records.

	Data collected
Flumazenil drug book	1. Reason for Flumazenil use
	2. Time of administration
	3. Time of discharge
Patient’s clinical record	1. Reason for Flumazenil use
	2. Time of administration
	3. Time of discharge

The standards for this reaudit cycle are mentioned in Table 2 and were based on guidelines mentioned in Table 3.

**Table 2:** The standards for this reaudit cycle.

The standards for this reaudit cycle
1. To write the justification of flumazenil use in the patient records as well as the flumazenil drug book (based on [1–4])
2. To write the timing of flumazenil use in the patient records as well as the Flumazenil drug book (based on [1, 2])
3. To write the discharge time in the patient’s record (based on [2, 5])

**Table 3:** The list of guidelines and recommendations that was considered for this re-audit.

Guidelines	Recommendations
Intercollegiate Advisory Committee for Sedation in Dentistry (IACSD) [8]	• Record of the clinical audit must be available for inspection • Regular high quality of clinical audit is compulsory for conscious sedation • Healthcare provider who involve in provision of CS should maintain high quality of record keeping i.e., patient’s written record or digital notes • Any adverse events or complication must be recorded through national system
Scottish Dental Clinical Effectiveness Programme (SDCEP) [1]	• Clinician should maintain high quality and up to date record keeping which include pre-sedation, peri-operative, monitoring phase and recovery phase • Time of drug administration and time of discharge must be recorded
General Dental Council, 2013 “Standards for the Dental Team” (GDC UK) [9]	Make and keep contemporaneous, complete and accurate patient records.
Faculty of General Dental Practice, United Kingdom [7] “Clinical examination & record keeping” FGDP, UK [9]	Patient’s record keeping must be up to date and accurate
Local standard by Sedation and Special Care Department (SSCD)	Based on first audit cycle and Guys and St Thomas Trust

## Results

The summary of the first audit cycle that was carried out for 12 weeks (2010) and the reaudit cycle in 2022 are shown in Table 4. Meanwhile, Table 5 illustrates the comparison of the reason for the use of Flumazenil between the years 2010 and 2022.

**Table 4:** The result summary of the first audit in 2010 and reaudit cycle in 2022.

Standards	Compliance (%) (1st Cycle)	Compliance (%) (2nd Cycle)
Standard 1:		
To write the justification of flumazenil use in the patient records and flumazenil drug book	100% (32/32)	100% (21/21)
Standard 2:		
To write the timing of flumazenil use in:		
1. Patient records	100% (32/32)	0% (0/21)
2. Flumazenil drug book	100% (32/32)	100% (0/21)
Standard 3:		
To write discharge time in the patient’s record	100% (32/32)	0% (0/21)

**Table 5:** Reason for Flumazenil use in SSCD in 2010 and 2022.

Reason for the use of Flumazenil (codes)	2010	2022
Code 1 = Emergency	0 (0%)	0 (0%)
Code 2 = Special care	8 (25%)	2 (9.5%)
Code 3 = Assist escort	1 (3%)	0 (0%)
Code 4 = Difficult journey	0 (0%)	1 (4.7%)
Code 5 = Prolong recovery	22 (69%)	9 (42.8%)
Code 6 = Post op retching	1 (3%)	0 (0%)
Code 7 = Others	0 (0%)	1 (4.7%)
Combine codes (1–7)	0 (0%)	8 (38%)

To improve the department’s CS record keeping, the findings of the audit were discussed at a department meeting with an agreed action plan. A stamp with a CS checklist was developed to be used in patient’s notes to ensure high-quality of record keeping. In Table 6, the result from the current CA in 2022 that was compared to the first cycle (2010) are displayed.

**Table 6:** Comparison the CA result for 2010 and 2022.

	2010		2022
Method of clinical audit	Prospective (fill in proforma)		Retrospective (Flumazenil Drug book and patient’s record)
Duration of audit	12 weeks (April 2019–June 2019)		24 weeks (January 2022–June 2022)
Total number of Midazolam given	453		665
Total number of Flumazenil given	32 (7%)		21 (3.15%)
Reasons for Flumazenil use	Emergency	0 (0%)	0 (0%)
	Special Care	8 (25%)	2 (9.5%)
	Assist escort	1 (3%)	0 (0%)
	Difficult journey	0 (0%)	1 (4.7%)
	Prolong recovery	22 (69%)	9 (42.8%)
	Post op retching	1 (3%)	0 (0%)
	Others	0 (0%)	1 (4.7%)
	Combine codes	0 (0%)	8 (38%)
Method of administration of Flumazenil	Intravenous (IV) = 31		Intravenous (IV) = 21
	• Increment dose = 21		
	• Bolus dose = 9		
	Intranasal (IN) = 1		
Time of sedation with Midazolam	Recorded		Not recorded
Time of Flumazenil administration	Recorded		Not recorded
Time of discharged	Recorded		Not recorded

**Fig. 2:**
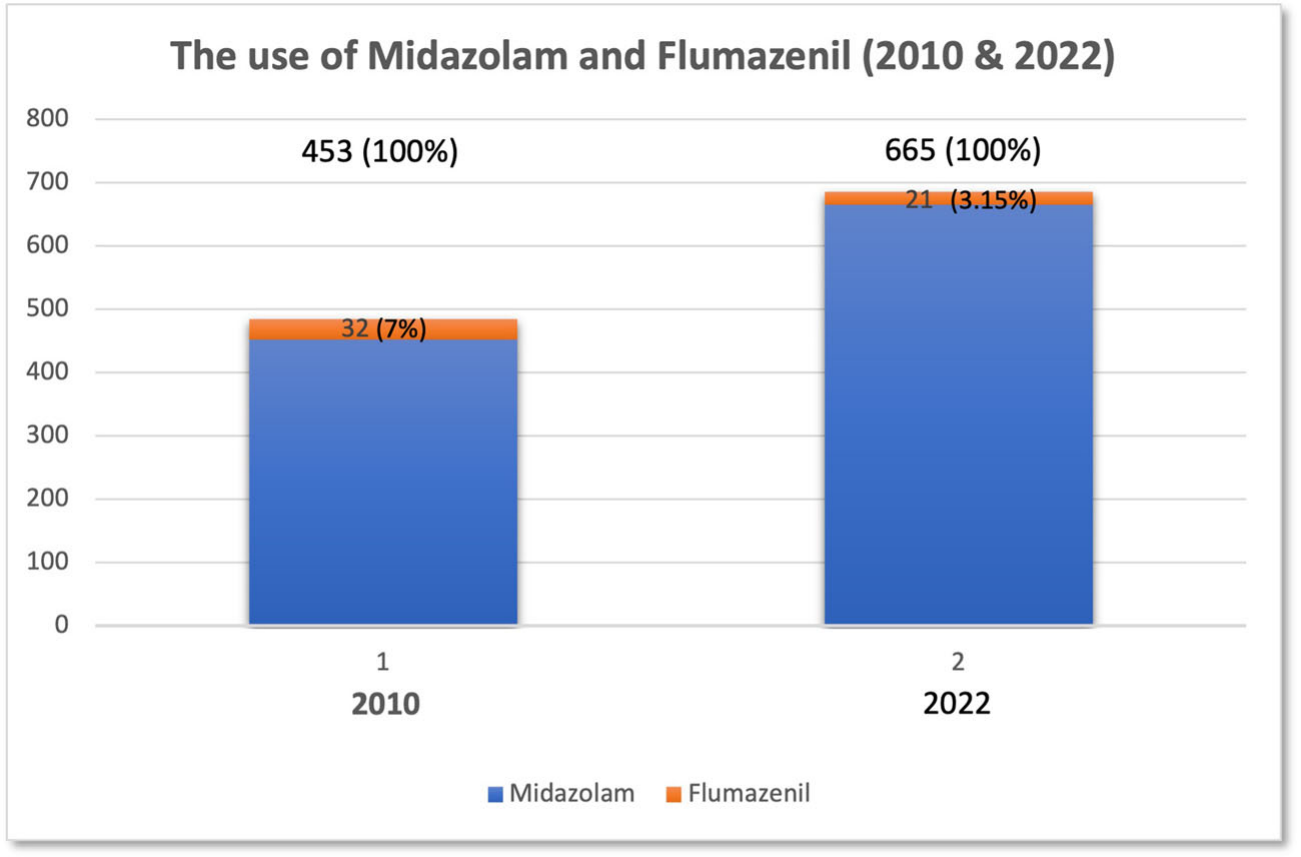
The use of IV midazolam and flumazenil in the Sedation and Special Care Department (SSCD).

**Fig. 3:**
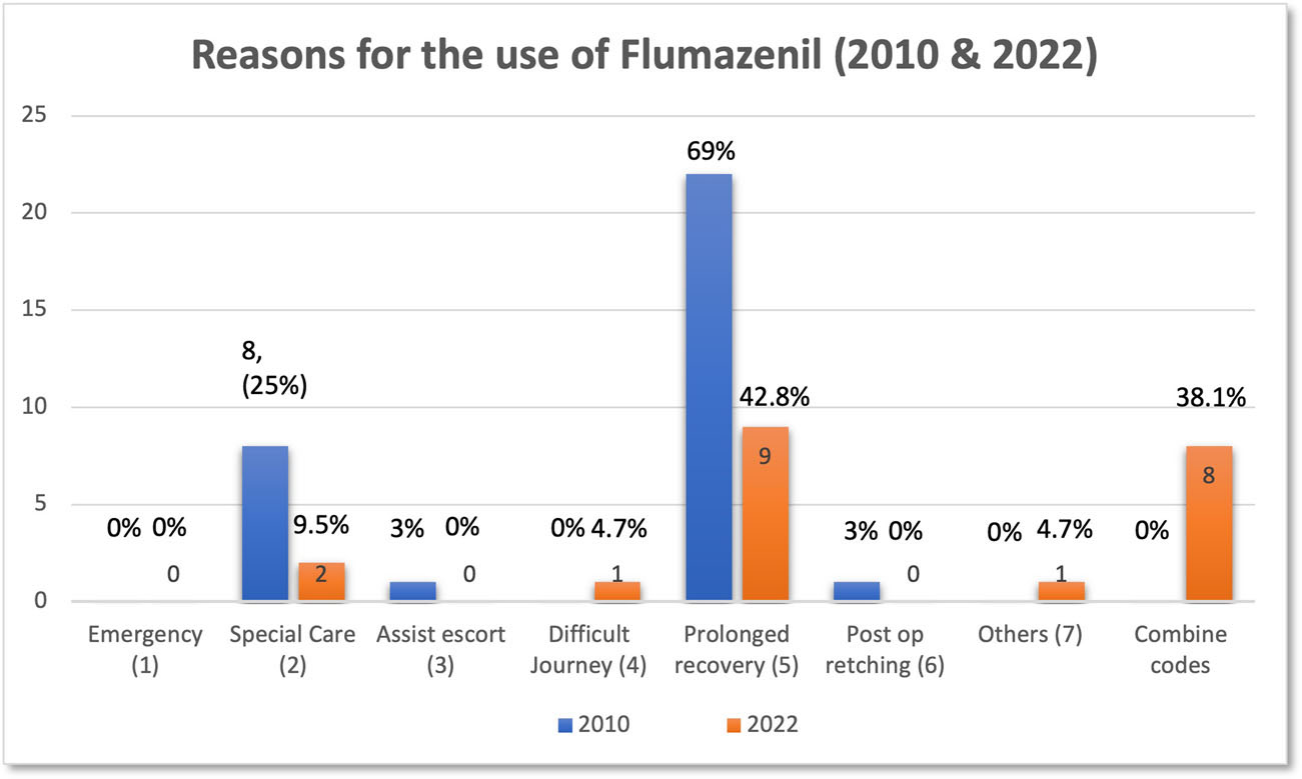
The reasons for flumazenil use (2010 and 2022).

There were a total of 665 patients who were given IV midazolam for dental procedures over the re-audit cycle (January to June 2022) in SSCD (Fig. 2). Out of 665 patients, 21 (3.1%) patients were given IV Flumazenil following a midazolam-induced CS procedure (Fig. 2). The justifications are shown in Fig. 3.

All clinicians (100%) did not document the time of midazolam administration in the patient’s clinical notes as recommended by IACSD and SDCEP guidelines and use of the Midazolam stamp checklist that was introduced during the first CA.

### The Flumazenil use

**Fig. 4:**
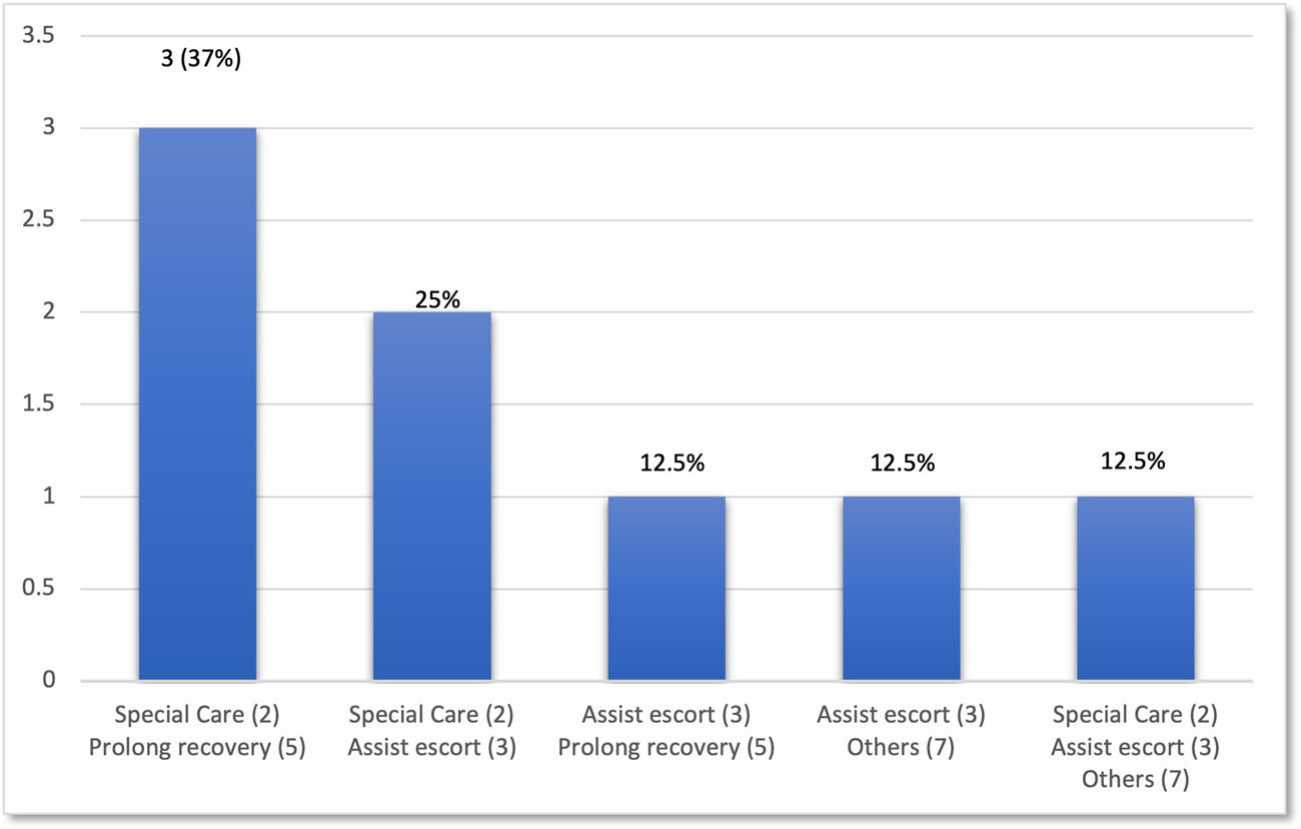
The reasons for flumazenil use (Combine codes).

During this period, none of the patients required flumazenil for emergency use and no post-operative retching following IV midazolam was recorded. Majority of the patients (9, 42.8%), had IV flumazenil due to prolonged recovery. In one case where the patient was a wheelchair user, flumazenil was administered to facilitate the patient’s journey back home (1, 4.7%). In 2 cases (9.5%), IV Flumazenil was administered because the patients had a learning disability diagnosis (autism, down syndrome and cerebral palsy). The other reason was to assist investigation procedure where the dental radiograph was needed to trace a missing bur that was lost during the dental procedure. In 8 cases (38%) a combination of several codes was used reflecting the complexity of the patient group seen in SSCD (Fig. 4).

All flumazenil (21, 100%) was given via IV cannula in contrast to the previous CA where flumazenil was given via both IV (31, 96.9%) and intranasal (IN) technique (1, 3.1%).

All clinicians (100%) had recorded the time of flumazenil administration in the flumazenil drug book however, this good practice was not recorded in the patient’s clinical notes as has been recommended by the IACSD and SDCEP (Table 4).

Following the administration of IV flumazenil and postoperative procedures, the patients will be discharged into the care of their escorts. Based on the current re-audit cycle, there was no discharge time recorded for all the patients that received IV flumazenil either in the flumazenil drug book or the information obtained from the patient’s clinical notes (21, 100%) (Table 4).

## Discussion

In the guidelines, it is recommended that CS services monitor the quality of their patient’s records and to ensure patient’s safety is guarded during dental treatment under CS. The available national guidelines outline approaches to improve the CS services’ quality and to obtain high-quality of record keeping. In order to monitor the effectiveness of existing clinical service, a CA (prospective or retrospective design) can be used. In a prospective CA design, the clinicians can design how to gather the desired information. This process can make the participants become aware of their procedures when they fill in a proforma to allow the auditor to capture the needed information. Meanwhile, a retrospective design can reflect what is actually happen but there might be less relevant information recorded and also the recorded data’s quality can be variable (e.g., data can be missing) [14].

The use of non-pharmacological and pharmacological interventions for patients is beneficial to reduce the anxiety level, especially among a group of patients where anxiety can trigger and/or worsen their medical conditions and in complex dental treatment. Although the administration of IV midazolam in SSCD has increased over the years, the use of IV flumazenil as reversal agent was low (3.5%) and comparatively similar to the first audit cycle in 2010 (7%) [6]. This audit has highlighted how the flumazenil following midazolam-induced CS can be based on the patient’s needs.

As shown in this audit, the number of occasions when flumazenil was used to address prolonged recovery (9, 42.8%) was comparable with the previous SSCD audit [6]. It is challenging to define exactly what normal recovery period is (for example one hour after the last given increment) following CS. This is why there are no explicit definitions identified in the literature. However, recovery from CS can be defined as ‘the phase from the end of treatment to a point when the patient is alert and comfortable. The patient can also present as psychological competent, orientated in time and space, and well ambulated’ [15]. Theoretically, the elimination half-life of midazolam (the length of time required for the concentration of midazolam to decrease to half of its starting dose in the body) is around 90 to180 min while the distribution half-life of midazolam (the time required for the plasma concentration to decline by 50%) can be anything from 6 to 20 min [16]. Therefore, it might be expected that the patients can show signs of recovery from 30 to 60 min after administration of the last titration of a sedative agent [5]. Nevertheless, there are other factors (such as the patient’s age, underlying medical history, route and speed of administration and the total dose of sedative agents itself) that can potentially impact the “normal recovery time” [17].

The clinicians should take a holistic view and evaluate the patient’s physical and psychological conditions before making any decision to administer IV flumazenil to enhance the recovery process [6]. Although the evidence is limited, it still indicates that some patients (e.g., patients with learning disability or neurological deficits) may experience reduced psychological and cognitive post procedure [18]. In this current audit, one case required IV flumazenil electively when the patient exhibited challenging behavior (such as removal of the cannula post procedure). In this circumstance, the use of IV flumazenil would be appropriate to prevent unwanted incidents and secure the safety of the patient, escort and the dental team.

The current CA highlighted the use of combination codes by the clinician reflecting the diverse patient cohorts seen in SSCD [4, 19]. Besides, this audit cycle has highlighted that using data that was recorded in the department’s flumazenil drug book can be limited. For example, the book recorded the patient’s name rather than the patient’s hospital number or NHS number. Recording a patient’s name can increased the risk of incorrect patient registration especially if the patients share similar surnames. A high quality clinical record keeping has accurate information that reflects the patient’s procedures in timely fashion [10].

The action plans recommendation are:

1. To design a tick box button in the new suggested electronic record-keeping software or a manual stamp checklist for paper-based clinical notes where the investigated relevant data (the time of midazolam administered, time of IV flumazenil given (if required) and time of discharge) is captured.
2. To arrange a friendly reminder for all the clinicians during department huddles in regard to maintaining high-quality record keeping.
3. To provide a refresher training for all new dental regarding record keeping

A period of six months for re-audit as regular clinical audits are recommended for monitoring the quality of records and compliance to the national requirements.

## Conclusion

Good record-keeping is essential as it allows inter-clinician communication and outlines the conversations that have taken place between the dental team (especially clinician) and the patient. A clinical audit is a quality improvement tool to aid in the assessment of current practice to the recommended standard. The current SSCD re-audit cycle was conducted to investigate the quality of record keeping and use of IV flumazenil in accordance with the recommendations made by national bodies (the NPSA’s RRR and the Faculty of General Dental Practice, UK).

## Data Availability

The authors confirm that the data supporting the findings of this study are available within the article [and/or] its supplementary materials.

## References

[1] (SDCEP) SDCEP. *Conscious sedation in Dentistry: Dental clinical guidance*. 2012; University of Dundee Dundee.

[2] Craig D, Boyle C. *Practical conscious sedation: Quintessenz Verlag*; 2019.

[3] McGloy R (1995). Reversal of conscious sedation by flumazenil: current status and future prospects. Acta Anaesthesiol Scand.

[4] Wilson C, Bird J, Harrison S. Dunning NA. Audit of flumazenil use in special care and oral surgery sedation services. Br Dent J. 2021. 10.1038/s41415-021-3001-4. 10.1038/s41415-021-3001-4 34045671

[5] Coulthard P, Sano K, Thomson P, Macfarlane T (2000). The effects of midazolam and flumazenil on psychomotor function and alertness in human volunteers. Br Dent J..

[6] Henthorn K, Dickinson C (2010). The use of flumazenil after midazolam-induced conscious sedation. Br Dent J..

[7] (IACSD) IACfSiD. Standards for Conscious Sedation in the Provision of Dental Care: Report of the Intercollegiate Advisory Committee for Sedation in Dentistry: RCS Publications; 2020.

[8] Kotecha R (2017). Clinical examination and record keeping: good practice guidelines, faculty of general dental practice United Kingdom(FGDP). Br Dent J..

[9] General Dental Council G. Standards for the Dental Team. https://www.gdc-uk.org/api/files/NEW%20Standards%20for%20the%20Dental%20Team.pdf 2013.

[10] Pullen I, Loudon J (2006). Improving standards in clinical record-keeping. Adv Psychiatr Treat..

[11] Bowie P, Bradley NA, Rushmer R (2012). Clinical audit and quality improvement—time for a rethink?. J Evaluat Clin Pract..

[12] Care Quality Commission C. Health and Social Care Act 2008 (Regulated Activities) Regulations 2014: Regulation 12: Safe care and treatment. *Care Quality Commission*; 2015.

[13] Brian Z, Weintraub JA. Peer Reviewed: Oral Health and COVID-19: Increasing the Need for Prevention and Access. *Preventing chronic disease*. 2020;17. 10.5888/pcd17.200266 PMC7458118 32790606

[14] Copeland G. A practical handbook for clinical audit. London: Clinical Governance Support Team, Department of Health Publications. 2005.

[15] Ogle OE, Hertz MB (2012). Anxiety control in the dental patient. Dent Clin..

[16] Kupietzky A, Houpt M (1993). Midazolam: a review of its use for conscious sedation in children. Pediatr Dent..

[17] Lugay M, Otto G, Kong M, Mason DJ, Wilets I (1996). Recovery time and safe discharge of endoscopy patients after conscious sedation. Gastroenterol. Nurs..

[18] Manley MCG, Skelly AM, Hamilton AG (2000). Dental treatment for people with challenging behaviour: general anaesthesia or sedation?. Br Dent J..

[19] Ransford N, Manley M, Lewis D, Thompson SA, Wray L, Boyle C (2010). Intranasal/intravenous sedation for the dental care of adults with severe disabilities: a multicentre prospective audit. Br Dent J..

